# Incidence and Characteristics of Cranial Nerve Injuries: A Nationwide Observational Study in Japan

**DOI:** 10.3390/jcm11164852

**Published:** 2022-08-18

**Authors:** Tomoya Hirose, Tetsuhisa Kitamura, Yusuke Katayama, Kenta Tanaka, Jotaro Tachino, Shunichiro Nakao, Kenichiro Ishida, Masahiro Ojima, Takeyuki Kiguchi, Yutaka Umemura, Kosuke Kiyohara, Jun Oda

**Affiliations:** 1 Department of Traumatology and Acute Critical Medicine, Graduate School of Medicine, Osaka University, Suita 565-0871, Japan; 2 Department of Social and Environmental Medicine, Division of Environmental Medicine and Population Sciences, Graduate School of Medicine, Osaka University, Suita 565-0871, Japan; 3 Department of Acute Medicine and Critical Care Center, Osaka National Hospital, National Hospital Organization, Osaka 540-0006, Japan; 4 Division of Trauma and Surgical Critical Care, Osaka General Medical Center, Osaka 558-8558, Japan; 5 Department of Food Science, Faculty of Home Economics, Otsuma Women’s University, Tokyo 102-8357, Japan

**Keywords:** cranial nerve, head trauma, facial trauma, injury, incidence, complication, brain injury, epidemiology, registry

## Abstract

**Background:** Large-scale data on cranial nerve injuries are scarce. **Methods**: This study enrolled 361,706 patients registered in the Japanese Trauma Data Bank from 2004 to 2018. We selected patients with cranial nerve injury using the corresponding Abbreviated Injury Scale codes and examined the incidence and characteristics. **Results:** In total, 347,101 patients were eligible for inclusion in our analysis. By mechanism of trauma, all cranial nerve injuries occurred in <1% of registered cases. The highest incidence was 0.2190% (55/25,117) for facial nerve injury in bicycle crash. By cause of trauma, all cranial nerve injuries occurred in <1% of registered cases. The highest incidence was 0.1943% (37/19,044) for facial nerve injury in occupational injury. No patients with spinal accessory nerve injury were observed. The most common cranial nerve injury was to the facial nerve (*n* = 278). Most cranial nerve injury patients are in the 30s to 50s age range, and there was a male predominance. Multiple cranial nerve injuries were observed in 81 patients. Many cranial nerve injury cases are complicated by skull base fractures. **Conclusions:** We revealed the incidence and characteristics of cranial nerve injury. Our findings may help physicians detect these injuries at an early stage in patients at risk.

## 1. Introduction

Injuries to the head or face are known to sometimes cause cranial nerve injuries [1]. These injuries are to the great detriment of the trauma patient in terms of their appearance and function. In many cases, cranial nerve injuries are not detected early and are recognized later, especially in cases of other trauma, such as life-threatening critical conditions or when the patient is under ventilatory management under anesthesia. Ideally, to detect these injuries at an early stage, the physician should quickly perform an assessment of the cranial nerve injury during the neurological survey at the emergency department for any patient who is at risk. In order for the physician to recognize this risk, it is necessary to identify and understand the incidence and characteristics that could result in cranial nerve injury.

Our review of the relevant literature revealed a fundamental scarcity of large-scale data on cranial nerve injuries in trauma patients. Several reports consisted of a few series concentrating on pediatric and adult head trauma patients with post-traumatic cranial nerve deficits [2,3]. Huckhagel et al. used the TraumaRegister DGU^®^ of the German Trauma Society to investigate cranial nerve injuries in patients with moderate to severe head trauma and concluded that cranial nerve injuries rarely occur in the context of traumatic brain injury (TBI), and that when present, they indicate a higher likelihood of functional impairment following primary care and that complicating skull base fractures should be suspected [4]. However, they did not examine trauma at the level of the individual cranial nerves (I: olfactory nerve—XII: hypoglossal nerve) in detail. To our knowledge, no previous studies have reported the incidence and characteristics of injuries to each cranial nerve.

The Japanese Trauma Data Bank (JTDB) is a nationwide trauma registry that is managed by the Japanese Association for the Surgery of Trauma (Trauma Surgery Committee) and the Japanese Association for Acute Medicine (Committee for Clinical Care Evaluation). Data registration in the JTDB was launched in 2003, and approximately 360,000 emergency patients with trauma were enrolled by 2018. The aim of the present study was to clarify the incidence and characteristics of injuries involving each cranial nerve in Japan using the JTDB registry.

## 2. Materials and Methods

### 2.1. Study Design and Settings

This was a retrospective observational study using the JTDB database. The study period spanned 15 years from January 2004 to December 2018. We included all emergency trauma patients registered in the JTDB database. We excluded patients with cardiopulmonary arrest on hospital arrival. Cardiopulmonary arrest on hospital arrival was defined as a systolic blood pressure of 0 mmHg and/or heart rate of 0 beats per minute on hospital arrival [5].

This study was approved by the Ethics Committee of the Osaka University Graduate School of Medicine (approval no. 16260). Personal identifiers were removed beforehand from the JTDB database, and thus the requirement for informed consent was waived. This study was written based on the STROBE statement to assess the reporting of cohort and cross-sectional studies [6].

### 2.2. Japanese Trauma Data Bank

The JTDB was launched in 2003 by the Japanese Association for the Surgery of Trauma (Trauma Surgery Committee) and the Japanese Association for Acute Medicine (Committee for Clinical Care Evaluation) [7]. In 2018, 280 major emergency medical institutions across Japan were registered in the JTDB database. These hospitals have an ability level equal to Level I trauma centers in the United States. Data from the participating institutions were collected via the Internet. In most cases, physicians and medical assistants who attended an abbreviated injury scale (AIS) coding course registered the data. The JTDB captures data on trauma patients, including age, sex, mechanism of injury, AIS code (version 1998), ISS, vital signs on hospital arrival, date and time series from hospital arrival to discharge, medical procedures (e.g., interventional radiology, surgical operations, and computed tomography scanning), and complications and mortality at discharge. ISS was calculated from the top three AIS scores in nine sites classified by AIS code.

We extracted the following patient data from the JTDB database: age, sex, cause of trauma, mechanism, ambulance type, systolic blood pressure, heart rate, respiratory rate, Glasgow Coma Scale score on hospital arrival, concomitant injury-AIS ≥ 3, concomitant injury-AIS ≥ 2, ISS, Trauma Injury Severity Score (TRISS), probability of survival (Ps) rate, in-hospital mortality, and AIS code of head trauma including cranial nerve injury. We selected patients with cranial nerve injury using the AIS codes shown in Table 1.

### 2.3. Analysis

Continuous variables are presented as the median and interquartile range, and categorical variables are presented as the number and percentage. The first step was to determine how many cranial nerve injuries occur in patients with head and facial trauma, by mechanism of trauma and by cause of trauma. Next, we examined the characteristics of trauma patients with cranial nerve injury. Finally, we clarified the head trauma and facial bone fracture complications that are found in patients with cranial nerve injury.

Descriptive statistics were calculated using JMP Pro 13 (SAS Institute Inc., Cary, NC, USA). We did not apply any statistical tests because of the nationwide population-based descriptive design of this study.

## 3. Results

**Figure 1:**
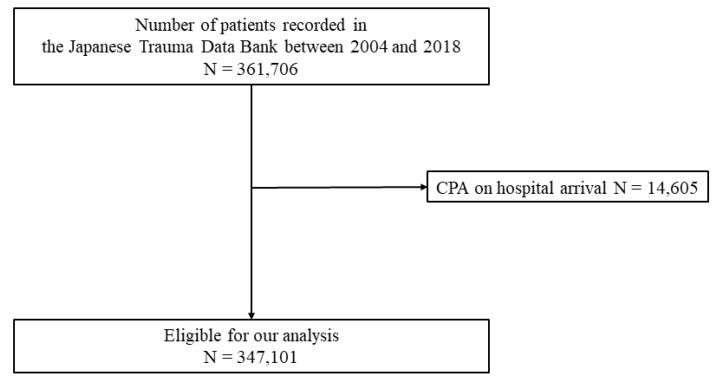
Patient flow. CPA, cardiopulmonary arrest.

Figure 1 shows the patient flow in this study. During the study period, 361,706 emergency patients were registered in the JTDB. We excluded patients with cardiopulmonary arrest on hospital arrival (*n* = 14,605). Finally, 347,101 patients were eligible for inclusion in our analysis.

### 3.1. Incidence of Cranial Nerve Injury

Table 2 shows the incidence of cranial nerve injuries in patients with head and facial trauma by mechanism of trauma and by cause of trauma.

By mechanism of trauma, all cranial nerve injuries occurred in <1% of registered cases. The highest incidence was 0.2190% (55/25,117) for facial nerve injury in bicycle crash. The next highest incidence was 0.2124% (76/35,782) for oculomotor nerve injury in motorcycle crash and 0.1792% (45/25,117) for optic nerve injury in bicycle crash.

By cause of trauma, all cranial nerve injuries occurred in <1% of registered cases. The highest incidence was 0.1943% (37/19,044) for facial nerve injury in occupational injury. The next highest incidence was 0.1103% (21/19,044) for optic nerve injury in occupational injury and 0.1084% (5/4614) for oculomotor nerve injury in injury.

In patients with head and facial trauma (AIS ≥ 2), all cranial nerve injuries occurred in <1%. The highest incidence was 0.6822% (148/21,693) for optic nerve injury in face injury. The next highest incidence was 0.4425% (96/21,693) for oculomotor nerve injury in face injury and 0.3596% (78/21,693) for facial nerve injury in face injury. Even in patients with head and facial trauma (AIS ≥ 3), all cranial nerve injuries occurred in <1%. The highest incidence was 0.8624% (21/2435) for oculomotor nerve injury in face injury. The next highest incidence was 0.8214% (20/2435) for optic nerve injury in face injury and 0.6160% (15/2435) for facial nerve injury in face injury.

### 3.2. Characteristics of Trauma Patients with Cranial Nerve Injury

Table 3 shows characteristics of trauma patients with cranial nerve injury. There were no registered cases of spinal accessory nerve injury. The most common cranial nerve injury was to the facial nerve (*n* = 278), followed by the oculomotor nerve (*n* = 261) and optic nerve (*n* = 245).

Most cranial nerve injury patients were in the 30s to 50s age range, and a male predominance was observed. Accidents were the most common cause, and blunt trauma, especially traffic accident, was the most common mechanism. Most patients were transported to the hospital by ambulance. There were few walk-in patients. Blood pressure, pulse, and respiratory rate were often stable, but many patients presented with mild to moderate consciousness disturbance. Many patients had a head injury of AIS ≥ 3 and facial trauma of AIS 2. The in-hospital mortality rates were low: 2.14% (5/234) in patients with optic nerve injury, 2.82% (7/248) in patients with oculomotor nerve injury, and 0% in patients with injury to other cranial nerves.

### 3.3. Head Trauma and Facial Bone Fracture Complicated with Cranial Nerve Injury

Table 4 shows the details of cases of head trauma and facial bone fracture complicated with cranial nerve injury. Multiple cranial nerve injuries were observed in 81 patients. Complications of brainstem injury were rare, and diffuse axonal injury was observed in six patients (2.3%, 6/230) with oculomotor nerve injury. Fewer cases with complicating cerebellar injury were seen in patients with optic nerve injury, oculomotor nerve injury, abducens nerve injury, and facial nerve injury. Complicating cerebral injury was observed in many cases. Only two cases of pituitary injury were seen, one in a patient with oculomotor nerve injury and one in a patient with abducens nerve injury. Complicating base fractures were seen in many cases, especially in patients with optic nerve injuries (54.29%, 133/245), abducens nerve injuries (50.88%, 29/57), facial nerve injuries (48.92%, 136/278), and auditory-vestibular nerve injuries (58.82%, 10/17). Complicating vault fractures were also seen in many cases, especially in patients with optic nerve injuries (34.69%, 85/245), facial nerve injuries (51.08%, 142/278), auditory-vestibular nerve injuries (58.82%, 10/17), and olfactory nerve injuries (43.75%, 7/16).

Facial bone complications were more common with maxillary, orbital, and zygomatic fractures than with mandibular fractures, especially optic nerve, oculomotor nerve, and trigeminal nerve injuries. LeFort II and III fractures were observed in 1 case (6.25%) with olfactory nerve injury, 17 cases (6.94%) with optic nerve injury, 14 cases (5.36%) with oculomotor nerve injury, 2 cases (9.52) with trigeminal nerve injury, 3 cases (5.26%) with abducens nerve injury, 2 cases (0.72%) with facial nerve injury, and none with other cranial nerve injuries.

## 4. Discussion

From the analysis of a nationwide trauma registry in Japan, this study revealed the incidence and characteristics of cranial nerve injuries. To our knowledge, this is the first report to reveal the incidence and characteristics of injury to each of the cranial nerves in a national-scale registry. Our findings not only provide basic epidemiological information about cranial nerve injury but will also help emergency department physicians quickly assess cranial nerve injury during a neurological survey for any patient at risk. In this study, the incidence rates of each cranial nerve injury by mechanism, by cause of trauma, and in patients with head and facial trauma (AIS ≥ 2 and AIS ≥ 3) were all <1% (Table 2). In a study of moderate-to-severe head trauma that used the TraumaRegister DGU^®^ of the German Trauma Society, Huckhagel et al. reported that cranial nerve injury was present in 1.0% of the total population [4]. Another study of a single hospital in China reported that that 9.1% of head trauma cases were complicated by cranial nerve injury [8]. The incidence rates of specific cranial nerve injuries reported from India are as follows: optic nerve, 2.78%; oculomotor, 2.9%; trochlear, 2.14%; abducens 3.02% [9]. The incidence of cranial nerve injury may be low in Japan. However, these studies are not reports of the incidence of cranial nerve injury in all trauma patients, as in our present study.

In 1992, Keane and Baloh reported that the olfactory, facial, and auditory-vestibular nerves are damaged most often, followed by the optic and ocular motor nerves, and that the trigeminal and lower cranial nerves are rarely involved [1]. This trend is approximately the same as that in our study; the most common cranial nerve injury was to the facial nerve (*n* = 278), followed by the oculomotor nerve (*n* = 261) and optic nerve (*n* = 245) (Table 3). Vital signs, such as blood pressure, pulse rate, and respiratory rate, were relatively stable, but the patients included those with a poor consciousness level at the time of arrival, probably due to the large number of head injury cases (Table 3). In general, the more severe the head injury is, the more likely it is to cause cranial nerve damage [4,10]. Cranial nerve injuries were identified in 9 (75%) of 12 autopsies of fatal traffic-related head trauma cases [11]. In the present study, the in-hospital mortality rate of trauma patients with cranial nerve injury was low (Table 3), which may be due to hidden cranial nerve injury that has not been detected in severe head trauma.

Table 4 shows head trauma and facial bone fracture complicated with cranial nerve injury. It is noteworthy that many cranial nerve injury cases are complicated by skull base fractures. Jacobi et al. reported that there was a link between the severity of the injury, fractures on the base of the skull, its foramina and channels, and the frequency of cranial nerve involvement in a long-term follow-up study of a pediatric head trauma cohort [2]. Huckhagel et al. reported that skull base fractures were significantly more frequent in cerebral nerve injury patients (51.0%) than in control cases (23.5%; *p* < 0.001), with an odds ratio of 3.38 (95% confidence interval 2.97–3.84) for cerebral nerve injury in a study of patients with moderate to severe head trauma [4]. Coello et al. recognized the predictive value of skull base fractures for a poor functional recovery in patients with cerebral nerve injury [3]. Therefore, when examining a patient with complicated skull base fracture, great care should be taken to ensure that the patient does not have concomitant cranial nerve trauma.

The present study was associated with some limitations. First, although we analyzed the JTDB database, in which major critical care centers in Japan participated, there were likely some selection biases because exhaustive research was not performed. Second, the diagnostic criteria for cranial nerve injury were based solely on the AIS codes in the JTDB. The diagnostic criteria for cranial nerve injury are not clear and depend on the evaluation at individual hospitals, and no information is available from the JTDB to provide a basis for the diagnosis of cranial nerve injury. We could not know who diagnosed and registered the cranial nerve injury or how many patients underwent CT or MRI scans or tracheotomy. Third, in patients with impaired consciousness, such as those with severe head trauma, cranial nerve damage may be missed. Fourth, the diagnosis of cranial nerve injury is assessed during the hospitalization period and therefore does not include the new emergence or disappearance of cranial nerve injury symptoms during long-term follow-up.

## 5. Conclusions

We revealed the incidence and characteristics of cranial nerve injuries using the JTDB. Our findings may help physicians detect these injuries at an early stage in patients at risk.

## Figures and Tables

**Table 1:** AIS codes of cranial nerve injury.

I Olfactory	II Optic	III Oculomotor	IV Trochlear	V Trigeminal	VI Abducens	VII Facial	VIII Auditory-Vestibular	IX Glossopharyngeal	X Vagus	XI Spinal Accessory	XII Hypoglo-ssal
130,499.2	130,699.2	130,899.2	131,099.2	131,299.2	131,499.2	131,699	131,899.2	132,099.2	132,299.2	132,499.2	132,699.2
130,402.2	130,602.2	130,802.2	131,002.2	131,202.2	131,402.2	131,602	131,802.2	132,002.2	132,202.2	132,402.2	132,602.2
130,404.2	130,604.2	130,804.2	131,004.2	131,204.2	131,404.2	131,604	131,804.2	132,004.2	132,204.2	132,404.2	132,604.2
	130,606.2						131,806.2				
	130,608.2										

AIS, Abbreviated Injury Scale.

**Table 2:** Incidence of cranial nerve injury.

	Mechanism, n (%)												
	Blunt—Traffic Accident	Blunt—Traffic Accident	Blunt—Traffic Accident	Blunt—Traffic Accident	Blunt—Traffic Accident	Blunt—Fall from an Elevation	Blunt—Fall from the Stairs	Blunt—Fall Down	Blunt—Sports	Blunt—Others	Penetrating	Others	Unknown
	Car Crash	Motorcycle Crash	Bicycle Crash	Pedestrian	Others								
	*n* = 36,088	*n* = 35,782	*n* = 25,117	*n* = 23,776	*n* = 1589	*n* = 28,858	*n* = 41,433	*n* = 95,710	*n* = 6766	*n* = 24,534	*n* = 11,556	*n* = 9518	*n* = 6374
**Cranial nerve injury**													
I Olfactory	0 (0)	4 (0.0112)	2 (0.0080)	4 (0.0168)	0 (0)	1 (0.0035)	2 (0.0048)	1 (0.0010)	1 (0.0148)	0 (0)	0 (0)	0 (0)	1 (0.0157)
II Optic	24 (0.0665)	50 (0.1397)	45 (0.1792)	18 (0.0757)	2 (0.1259)	31 (0.1074)	32 (0.0772)	20 (0.0209)	3 (0.0443)	7 (0.0285)	9 (0.0779)	0 (0)	4 (0.0628)
III Oculomotor	39 (0.1081)	76 (0.2124)	44 (0.1752)	31 (0.1303)	0 (0)	28 (0.0970)	14 (0.0338)	6 (0.0063)	6 (0.0887)	12 (0.0489)	0 (0)	0 (0)	5 (0.0784)
IV Trochlear	7 (0.0194)	4 (0.0112)	2 (0.0080)	1 (0.0042)	0 (0)	0 (0)	1 (0.0024)	2 (0.0021)	0 (0)	1 (0.0041)	0 (0)	0 (0)	0 (0)
V Trigeminal	2 (0.0055)	5 (0.0140)	3 (0.0119)	1 (0.0042)	0 (0)	3 (0.0104)	2 (0.0048)	1 (0.0010)	1 (0.0148)	3 (0.0122)	0 (0)	0 (0)	0 (0)
VI Abducens	11 (0.0305)	7 (0.0196)	4 (0.0159)	9 (0.0379)	0 (0)	8 (0.0277)	7 (0.0169)	2 (0.0021)	1 (0.0148)	6 (0.0245)	1 (0.0087)	0 (0)	1 (0.0157)
VII Facial	19 (0.0526)	59 (0.1649)	55 (0.2190)	20 (0.0841)	2 (0.1259)	31 (0.1074)	36 (0.0869)	16 (0.0167)	3 (0.0443)	26 (0.1060)	10 (0.0865)	0 (0)	1 (0.0157)
VIII Auditory-vestibular	2 (0.0055)	3 (0.0084)	5 (0.0199)	2 (0.0084)	0 (0)	2 (0.0069)	1 (0.0024)	1 (0.0010)	0 (0)	1 (0.0041)	0 (0)	0 (0)	0 (0)
IX Glossopharyngeal	1 (0.0028)	0 (0)	0 (0)	0 (0)	0 (0)	0 (0)	0 (0)	0 (0)	0 (0)	0 (0)	1 (0.0087)	0 (0)	0 (0)
X Vagus	0 (0)	0 (0)	0 (0)	1 (0.0042)	0 (0)	0 (0)	0 (0)	0 (0)	0 (0)	0 (0)	0 (0)	0 (0)	0 (0)
XI Spinal Accessory	0 (0)	0 (0)	0 (0)	0 (0)	0 (0)	0 (0)	0(0)	0 (0)	0 (0)	0 (0)	0 (0)	0 (0)	0 (0)
XII Hypoglossal	0 (0)	0 (0)	0 (0)	0 (0)	0 (0)	0 (0)	1 (0.0024)	0 (0)	0 (0)	0 (0)	0 (0)	0 (0)	0 (0)
	**Concomitant injury—AIS** **≥ 3, *n* (%)**			**Concomitant injury—AIS** **≥ 2, *n* (%)**			**Cause of trauma, *n* (%)**						
	Head injury		Face injury	Head injury		Face injury	Accident		Suicide	Injury	Occupational injury	Others	Unknown
	*n* = 96,244		*n* = 2435	*n* = 110,464		*n* = 21,693	*n* = 292,793		*n* = 16,810	*n* = 4614	*n* = 19,044	*n* = 3659	*n* = 10,181
**Cranial nerve injury**													
I Olfactory	14 (0.0145)		0 (0)	16 (0.0145)		3 (0.0138)	14 (0.0048)		1 (0.0059)	0 (0)	0 (0)	0 (0)	1 (0.0098)
II Optic	212 (0.2203)		20 (0.8214)	245 (0.2218)		148 (0.6822)	205 (0.0700)		10 (0.0595)	2 (0.0433)	21 (0.1103)	1 (0.0273)	6 (0.0589)
III Oculomotor	212 (0.2203)		21 (0.8624)	261 (0.2363)		96 (0.4425)	233 (0.0796)		7 (0.0416)	5 (0.1084)	11 (0.0578)	1 (0.0273)	4 (0.0393)
IV Trochlear	14 (0.0145)		1 (0.0411)	18 (0.0163)		4 (0.0184)	17 (0.0058)		0 (0)	0 (0)	1 (0.0053)	0 (0)	0 (0)
V Trigeminal	13 (0.0135)		3 (0.1232)	21 (0.0190)		14 (0.0645)	18 (0.0061)		2 (0.0119)	0 (0)	1 (0.0053)	0 (0)	0 (0)
VI Abducens	50 (0.0520)		2 (0.0821)	57 (0.0462)		21 (0.0968)	45 (0.0154)		1 (0.0059)	1 (0.0217)	10 (0.0525)	0 (0)	0 (0)
VII Facial	230 (0.2390)		15 (0.6160)	278 (0.2517)		78 (0.3596)	227 (0.0775)		6 (0.0357)	4 (0.0867)	37 (0.1943)	1 (0.0273)	3 (0.0295)
VIII Auditory-vestibular	16 (0.0166)		0 (0)	17 (0.0154)		4 (0.0184)	15(0.0051)		0 (0)	0 (0)	1 (0.0053)	0 (0)	1 (0.0098)
IX Glossopharyngeal	1 (0.0010)		0 (0)	2 (0.0018)		0 (0)	1 (0.0003)		1 (0.0059)	0 (0)	0 (0)	0 (0)	0 (0)
X Vagus	1 (0.0010)		0 (0)	1 (0.0009)		0 (0)	1 (0.0003)		0 (0)	0 (0)	0 (0)	0 (0)	0 (0)
XI Spinal Accessory	0 (0)		0 (0)	0 (0)		0 (0)	0 (0)		0 (0)	0 (0)	0 (0)	0 (0)	0 (0)
XII Hypoglossal	1 (0.0010)		0 (0)	1 (0.0009)		0 (0)	1 (0.0003)		0 (0)	0 (0)	0 (0)	0 (0)	0 (0)

AIS, Abbreviated Injury Scale.

**Table 3:** Characteristics of trauma patients with cranial nerve injury.

	I Olfactory	II Optic	III Oculomotor	IV Trochlear	V Trigeminal	VI Abducens	VII Facial	VIII Auditory-Vestibular	IX Glossopharyngeal	X Vagus	XI Spinal Accessory	XII Hypoglossal
	*n* = 16	*n* = 245	*n* = 261	*n* = 18	*n* = 21	*n* = 57	*n* = 278	*n* = 17	*n* = 2	*n* = 1	*n* = 0	*n* = 1
**Age, years, median (IQR)**	33 (19.5–58.75)	45 (25–61.5)	47 (29.5–63)	55 (26.75–66.25)	31 (22–48)	43 (27.5–59.5)	41 (24–56)	42 (19–62.5)	49.5 (33–66)	73 (73–73)	N/A	71 (71–71)
**Male Sex, *n* (%)**	9 (56.25)	197 (80.41)	156 (59.77)	10 (55.56)	16 (76.19)	37 (64.91)	194 (69.78)	13 (76.47)	2 (100)	0 (0)	N/A	1 (100)
**Cause of trauma, *n* (%)**												
Accident	14 (87.5)	205 (83.67)	233 (89.27)	17 (94.44)	18 (85.71)	45 (78.95)	227 (81.65)	15 (88.24)	1 (50)	1 (100)	N/A	1 (100)
Suicide	1 (6.25)	10 (4.08)	7 (2.68)	0 (0)	2 (9.52)	1 (1.75)	6 (2.16)	0 (0)	1 (50)	0 (0)	N/A	0 (0)
Injury	0 (0)	2 (0.82)	5 (1.92)	0 (0)	0 (0)	1 (1.75)	4 (1.44)	0 (0)	0 (0)	0 (0)	N/A	0 (0)
Occupational injury	0 (0)	21 (8.57)	11 (4.21)	1 (5.56)	1 (4.76)	10 (17.54)	37 (13.31)	1 (5.88)	0 (0)	0 (0)	N/A	0 (0)
Others	0 (0)	1 (0.41)	1 (0.38)	0 (0)	0 (0)	0 (0)	1 (0.36)	0 (0)	0 (0)	0 (0)	N/A	0 (0)
Unknown	1 (6.25)	6 (2.45)	4 (1.53)	0 (0)	0 (0)	0 (0)	3 (1.08)	1 (5.88)	0 (0)	0 (0)	N/A	0 (0)
**Mechanism, *n* (%)**												
Blunt- Traffic accident												
Car crash	0 (0)	24 (9.8)	39 (14.94)	7 (38.89)	2 (9.52)	11 (19.30)	19 (6.83)	2 (11.76)	1 (50)	0 (0)	N/A	0 (0)
Motorcycle crash	4 (25)	50 (20.41)	76 (29.12)	4 (22.22)	5 (23.81)	7 (12.28)	59 (21.22)	3 (17.65)	0 (0)	0 (0)	N/A	0 (0)
Bicycle crash	2 (12.5)	45 (18.37)	44 (16.86)	2 (11.11)	3 (14.29)	4 (7.02)	55 (19.78)	5 (29.41)	0 (0)	0 (0)	N/A	0 (0)
Pedestrian	4 (25)	18 (7.35)	31 (11.88)	1 (5.56)	1 (4.76)	9 (15.79)	20 (7.19)	2 (11.76)	0 (0)	1 (100)	N/A	0 (0)
Others	0 (0)	2 (0.82)	0 (0)	0 (0)	0 (0)	0 (0)	2 (0.72)	0 (0)	0 (0)	0 (0)	N/A	0 (0)
Blunt—Fall from an elevation	1 (6.25)	31 (12.65)	28 (10.73)	0 (0)	3 (14.29)	8 (14.04)	31 (11.15)	2 (11.76)	0 (0)	0 (0)	N/A	0 (0)
Blunt—Fall from the stairs	2 (12.5)	32 (13.06)	14 (5.36)	1 (5.56)	2 (9.52)	7 (12.28)	36 (12.95)	1 (5.88)	0 (0)	0 (0)	N/A	1 (100)
Blunt—Fall down	1 (6.25)	20 (8.16)	6 (2.30)	2 (11.11)	1 (4.76)	2 (3.51)	16 (5.76)	1 (5.88)	0 (0)	0 (0)	N/A	0 (0)
Blunt—Sports	1 (6.25)	3 (1.22)	6 (2.30)	0 (0)	1 (4.76)	1 (1.75)	3 (1.08)	0 (0)	0 (0)	0 (0)	N/A	0 (0)
Blunt—Others	0 (0)	7 (2.86)	12 (4.60)	1 (11.11)	3 (18.14)	6 (10.53)	26 (9.35)	1 (5.88)	0 (0)	0 (0)	N/A	0 (0)
Penetrating	0 (0)	9 (3.67)	0 (0)	0 (0)	0 (0)	1 (1.75)	10 (3.6)	0 (0)	1 (50)	0 (0)	N/A	0 (0)
Others	0 (0)	0 (0)	0 (0)	0 (0)	0 (0)	0 (0)	0 (0)	0 (0)	0 (0)	0 (0)	N/A	0 (0)
Unknown	1 (6.25)	4 (1.63)	5 (1.92)	0 (0)	0 (0)	1 (1.75)	1 (0.36)	0 (0)	0 (0)	0 (0)	N/A	0 (0)
	**I Olfactory**	**II Optic**	**III Oculomotor**	**IV Trochlear**	**V Trigeminal**	**VI Abducens**	**VII Facial**	**VIII Auditory-Vestibular**	**IX Glossopharyngeal**	**X Vagus**	**XI Spinal Accessory**	**XII Hypoglossal**
	***n* = 16**	***n* = 245**	***n* = 261**	***n* = 18**	***n* = 21**	***n* = 57**	***n* = 278**	***n* = 17**	***n* = 2**	***n* = 1**	***n* = 0**	***n* = 1**
**Ambulance type**												
Ordinary ambulance	15 (93.75)	211 (86.12)	194 (74.33)	12 (66.67)	17 (80.95)	41 (71.93)	231 (83.09)	15 (88.24)	1 (50)	1 (100)	N/A	1 (100)
Physician-staffed helicopter	0 (0)	16 (6.53)	25 (9.58)	3 (16.67)	1 (4.76)	12 (21.05)	23 (8.27)	0 (0)	0 (0)	0 (0)	N/A	0 (0)
Physician-staffed ambulance	0 (0)	7 (2.86)	35 (13.41)	1 (5.56)	1 (4.76)	0 (0)	10 (3.6)	0 (0)	1 (50)	0 (0)	N/A	0 (0)
Walk-in	1 (6.25)	5 (2.04)	0 (0)	1 (5.56)	1 (4.76)	2 (3.51)	4 (14.4)	2 (11.76)	0 (0)	0 (0)	N/A	0 (0)
Others	0 (0)	2 (0.82)	3 (1.15)	0 (0)	0 (0)	1 (1.75)	2 (0.72)	0 (0)	0 (0)	0 (0)	N/A	0 (0)
Unknown	0 (0)	4 (1.63)	4 (1.53)	1 (5.56)	1 (4.76)	1 (1.75)	8 (2.88)	0 (0)	0 (0)	0 (0)	N/A	0 (0)
**Systolic BP on arrival, mmHg, median (IQR)**	135 (119–155) (*n* = 15)	136 (119–154) (*n* = 239)	135 (118–150.8) (*n* = 260)	142.5 (124.5–150)	130 (114.5–149)	131 (114–151)	132 (114–150) (*n* = 274)	131 (126.5–142)	148 (110–186)	145 (145–145)	*n*/A	156 (156–156)
**Heart rate on arrival, bpm, median (IQR)**	90 (70–96) (*n* = 15)	82 (69–98) (*n* = 234)	86 (73–98) (*n* = 259)	82 (71.75–93)	73 (69–87)	90 (75.5–110.5)	87 (76–98) (*n* = 271)	76 (70–90.5)	85.5 (70–101)	89 (89–89)	N/A	97 (97–97)
**Respiratory rate on arrival, median (IQR)**	18 (16–22.5) (*n* = 14)	20 (18–24) (*n* = 219)	21 (18–25) (*n* = 247)	19.5 (16–24) (*n* = 16)	20 (18–21) (*n* = 19)	21 (18–279 (*n* = 53)	20 (18–24) (*n* = 256)	20 (17–22)	21.5 (16–27)	16 (16–16)	N/A	19 (19–19)
**GCS score on arrival, median (IQR)**	14 (13–15) (*n* = 15)	14 (11–15) (*n* = 236)	13 (8–14) (*n* = 257)	13 (11.75–14.25)	14 (9–15)	14 (11–15)	13 (10–15) (*n* = 267)	14 (12.5–15)	13 (13–13)	14 (14–14)	N/A	13 (13–13)
**Concomitant injury—AIS** **≥ 3**												
Head injury, *n* (%)	14 (87.5)	212 (86.53)	212 (81.23)	14 (77.78)	13 (61.9)	50 (85.72)	230 (82.73)	16 (94.12)	1 (50)	1 (100)	N/A	1 (100)
Face injury, *n* (%)	0 (0)	20 (8.16)	21 (8.05)	1 (5.56)	3 (14.29)	2 (3.51)	15 (5.4)	0 (0)	0 (0)	0 (0)	N/A	0 (0)
Neck injury, *n* (%)	0 (0)	2 (0.82)	3 (1.15)	0 (0)	0 (0)	0 (0)	2 (0.72)	0 (0)	1 (50)	0 (0)	N/A	0 (0)
Chest injury, *n* (%)	3 (18.75)	61 (24.9)	90 (34.48)	5 (27.78)	2 (9.52)	19 (33.33)	72 (25.9)	1 (5.88)	1 (50)	0 (0)	N/A	0 (0)
Abdominal injury, *n* (%)	0 (0)	9 (3.67)	14 (5.36)	0 (0)	0 (0)	2 (3.51)	8 (2.88)	0 (0)	0 (0)	0 (0)	N/A	0 (0)
Spine injury, *n* (%)	0 (0)	6 (2.45)	12 (4.6)	0 (0)	0 (0)	3 (5.26)	6 (2.16)	0 (0)	0 (0)	0 (0)	N/A	0 (0)
Lower extremity injury, *n* (%)	2 (12.5)	28 (11.43)	46 (17.62)	2 (0.77)	0 (0)	5 (8.77)	17 (6.12)	0 (0)	0 (0)	0 (0)	N/A	0 (0)
Upper extremity injury, *n* (%)	0 (0)	17 (6.94)	13 (4.98)	0 (0)	1 (4.76)	1 (1.75)	4 (1.44)	2 (11.76)	0 (0)	0 (0)	N/A	0 (0)
	**I Olfactory**	**II Optic**	**III Oculomotor**	**IV Trochlear**	**V Trigeminal**	**VI Abducens**	**VII Facial**	**VIII Auditory-Vestibular**	**IX Glossopharyngeal**	**X Vagus**	**XI Spinal Accessory**	**XII Hypoglossal**
	***n* = 16**	***n* = 245**	***n* = 261**	***n* = 18**	***n* = 21**	***n* = 57**	***n* = 278**	***n* = 17**	***n* = 2**	***n* = 1**	***n* = 0**	***n* = 1**
**Concomitant injury—AIS** **≥ 2**												
Head injury, *n* (%)	15 (93.75)	217 (88.57)	229 (87.74)	17 (94.44)	13 (61.9)	50 (85.72)	254 (91.37)	17 (100)	1 (50)	0 (0)	N/A	1 (100)
Face injury, *n* (%)	3 (18.75)	148 (60.41)	96 (36.78)	4 (22.22)	14 (66.67)	21 (36.84)	78 (28.06)	4 (23.53)	0 (0)	0 (0)	N/A	0 (0)
Neck injury, *n* (%)	0 (0)	2 (0.82)	3 (1.15)	0 (0)	0 (0)	0 (0)	5 (1.80)	0 (0)	1 (50)	0 (0)	N/A	0 (0)
Chest injury, *n* (%)	3 (18.75)	69 (28.16)	99 (37.93)	5 (27.78)	3 (14.29)	20 (35.09)	75 (26.98)	1 (5.88)	1 (50)	1 (100)	N/A	0 (0)
Abdominal injury, *n* (%)	1 (6.25)	20 (8.16)	31 (11.88)	3 (16.67)	0 (0)	4 (7.02)	21 (7.55)	0 (0)	1 (50)	0 (0)	N/A	0 (0)
Spine injury, *n* (%)	0 (0)	26 (10.61)	38 (14.56)	3 (16.67)	2 (9.52)	8 (14.04)	26 (9.35)	2 (11.76)	1 (50)	0 (0)	N/A	0 (0)
Lower extremity injury, *n* (%)	3 (18.75)	50 (20.41)	78 (29.89)	3 (16.67)	3 (14.29)	15 (26.32)	44 (15.83)	1 (5.88)	0 (0)	0 (0)	N/A	0 (0)
Upper extremity injury, *n* (%)	0 (0)	63 (25.71)	73 (27.97)	4 (22.22)	4 (19.05)	11 (19.3)	66 (23.74)	2 (11.76)	1 (50)	1 (100)	N/A	0 (0)
**ISS, median (IQR)**	12 (9.25–17)	21 (14–29)	25 (14–34)	20.5 (9–31)	14 (9–21.5)	21 (14–29)	17 (13–25.25)	17 (10–21)	21 (20–22)	17 (17–17)	N/A	10 (10–10)
**TRISS probability of survival, median (IQR)**	0.98 (0.92–0.99) (*n* = 13)	0.96 (0.87–0.98) (*n* = 204)	0.92 (0.81–0.98) (*n* = 241)	0.96 (0.83–0.97) (*n* = 16)	0.98 (0.95–0.99) (*n* = 19)	0.96 (0.88–0.98) (*n* = 52)	0.96 (0.92–0.99) (*n* = 249)	0.98 (0.95- 0.99)	0.96 (0.94–0.98)	0.94 (0.94–0.94)	N/A	0.96 (0.96–0.96)
**In-hospital mortality, *n* (%)**	0 (0)	5 (2.14) (*n* = 234)	7 (2.82) (*n* = 248)	0 (0)	0 (0) (*n* = 20)	0 (0) (*n* = 56)	0 (0) (*n* = 266)	0 (0)	0 (0)	0 (0)	N/A	0 (0)

AIS; Abbreviated Injury Scale, GCS; Glasgow Coma Scare, ISS; Injury severity score, IQR; Interquartile range, TRISS; Trauma injury severity score.

**Table 4:** Head trauma and facial bone fracture complicated with cranial nerve injury.

	I Olfactory	II Optic	III Oculomotor	IV Trochlear	V Trigeminal	VI Abducens	VII Facial	VIII Auditory-Vestibular	IX Glossopharyngeal	X Vagus	XI Spinal Accessory	XII Hypoglossal
	*n* = 16	*n* = 245	*n* = 261	*n* = 18	*n* = 21	*n* = 57	*n* = 278	*n* = 17	*n* = 2	*n* = 1	*n* = 0	*n* = 1
**Cranial nerve injury, *n*, %**												
I Olfactory	-	1 (0.41)	0 (0)	0 (0)	0 (0)	0 (0)	2 (0.72)	0 (0)	0 (0)	0 (0)	N/A	0 (0)
II Optic	1 (6.25)	-	10 (3.83)	1 (5.56)	3 (14.29)	3 (5.26)	5 (1.80)	1 (5.88)	0 (0)	0 (0)	N/A	0 (0)
III Oculomotor	0 (0)	10 (4.08)	-	6 (33.33)	4 (19.05)	9 (15.79)	12 (4.32)	0 (0)	0 (0)	0 (0)	N/A	0 (0)
IV Trochlear	0 (0)	1 (0.41)	6 (2.30)	-	0 (0)	0 (0)	1 (0.36)	0 (0)	0 (0)	0 (0)	N/A	0 (0)
V Trigeminal	0 (0)	3 (1.22)	4 (1.53)	0 (0)	-	1 (1.75)	3 (1.08)	0 (0)	0 (0)	0 (0)	N/A	0 (0)
VI Abducens	0 (0)	3 (1.22)	9 (3.45)	0 (0)	1 (4.76)	-	11 (3.96)	1 (5.88)	0 (0)	0 (0)	N/A	0 (0)
VII Facial	2 (12.50)	5 (2.04)	12 (4.60)	1 (5.56)	3 (14.29)	11 (19.3)	-	6 (35.29)	0 (0)	0 (0)	N/A	1 (100)
VIII Auditory-vestibular	0 (0)	1 (0.41)	0 (0)	0 (0)	0 (0)	1 (1.75)	6 (2.16)	-	0 (0)	0 (0)	N/A	0 (0)
IX Glossopharyngeal	0 (0)	0 (0)	0 (0)	0 (0)	0 (0)	0 (0)	0 (0)	0 (0)	-	0 (0)	N/A	0 (0)
X Vagus	0 (0)	0 (0)	0 (0)	0 (0)	0 (0)	0 (0)	0 (0)	0 (0)	0 (0)	-	N/A	0 (0)
XI Spinal Accessory	0 (0)	0 (0)	0 (0)	0 (0)	0 (0)	0 (0)	0 (0)	0 (0)	0 (0)	0 (0)	-	0 (0)
XII Hypoglossal	0 (0)	0 (0)	0 (0)	0 (0)	0 (0)	0 (0)	1 (100)	0 (0)	0 (0)	0 (0)	N/A	-
**Brain stem, *n*, %**												
Injury involving hemorrhage	0 (0)	0 (0)	0 (0)	0 (0)	0 (0)	1 (1.75)	0 (0)	0 (0)	0 (0)	0 (0)	N/A	0 (0)
Diffuse axonal injury	0 (0)	0 (0)	6 (2.30)	0 (0)	0 (0)	0 (0)	0 (0)	0 (0)	0 (0)	0 (0)	N/A	0 (0)
**Cerebellum, *n*, %**												
Subarachnoid hemorrhage	0 (0)	0 (0)	2 (0.77)	0 (0)	0 (0)	0 (0)	1 (0.36)	0 (0)	0 (0)	0 (0)	N/A	0 (0)
Epidural or extradural hematoma	0 (0)	1 (0.41)	0 (0)	0 (0)	0 (0)	0 (0)	1 (0.36)	0 (0)	0 (0)	0 (0)	N/A	0 (0)
Subdural hematoma	0 (0)	0 (0)	1 (0.38)	0 (0)	0 (0)	1 (1.75)	1 (0.36)	0 (0)	0 (0)	0 (0)	N/A	0 (0)
Subpial hemorrhage	0 (0)	1 (0.41)	0 (0)	0 (0)	0 (0)	0 (0)	0 (0)	0 (0)	0 (0)	0 (0)	N/A	0 (0)
Contusion	0 (0)	0 (0)	1 (0.38)	0 (0)	0 (0)	1 (1.75)	0 (0)	0 (0)	0 (0)	0 (0)	N/A	0 (0)
Intracerebellar including petechial and subcortical	0 (0)	0 (0)	0 (0)	0 (0)	0 (0)	0 (0)	1 (0.36)	0 (0)	0 (0)	0 (0)	N/A	0 (0)
	**I Olfactory**	**II Optic**	**III Oculomotor**	**IV Trochlear**	**V Trigeminal**	**VI Abducens**	**VII Facial**	**VIII Auditory-Vestibular**	**IX Glossopharyngeal**	**X Vagus**	**XI Spinal Accessory**	**XII Hypoglossal**
	***n* = 16**	***n* = 245**	***n* = 261**	***n* = 18**	***n* = 21**	***n* = 57**	***n* = 278**	***n* = 17**	***n* = 2**	***n* = 1**	***n* = 0**	***n* = 1**
**Cerebrum, *n*, %**												
Subarachnoid hemorrhage	8 (50)	83 (33.88)	149 (57.09)	11 (61.11)	5 (23.81)	22 (38.60)	127 (45.68)	6 (35.29)	0 (0)	1 (100)	N/A	1 (100)
Pneumocephalus	2 (12.5)	67 (27.62)	22 (8.43)	1 (5.56)	4 (19.05)	20 (35.09)	52 (18.71)	2 (11.76)	0 (0)	0 (0)	N/A	0 (0)
Epidural or extradural hematoma	0 (0)	56 (22.86)	35 (13.41)	1 (5.56)	4 (19.05)	9 (15.79)	38 (13.67)	1 (5.88)	0 (0)	0 (0)	N/A	0 (0)
Intracerebral hematoma	0 (0)	5 (2.04)	8 (3.07)	0 (0)	0 (0)	1 (1.75)	1 (0.36)	0 (0)	0 (0)	0 (0)	N/A	0 (0)
Subdural hematoma	2 (12.5)	63 (25.71)	57 (21.84)	3 (16.67)	3 (14.29)	17 (29.82)	73 (26.26)	8 (47.06)	0 (0)	0 (0)	N/A	0 (0)
Intraventricular hemorrhage	0 (0)	7 (2.86)	9 (3.45)	0 (0)	0 (0)	1 (1.75)	3 (1.08)	0 (0)	0 (0)	0 (0)	N/A	0 (0)
Contusion	12 (75)	80 (32.65)	88 (33.72)	7 (38.89)	6 (28.57)	17 (29.82)	122 (43.88)	7 (41.18)	0 (0)	1 (100)	N/A	1 (100)
Diffuse axonal injury	0 (0)	21 (8.57)	49 (18.77)	4 (22.22)	0 (0)	3 (5.26)	20 (7.19)	1 (5.88)	0 (0)	0 (0)	N/A	0 (0)
**Pituitary injury, *n*, %**	0 (0)	0 (0)	1 (0.38)	0 (0)	0 (0)	1 (1.75)	0 (0)	0 (0)	0 (0)	0 (0)	N/A	0 (0)
**Base fracture, *n*, %**	5 (31.25)	133 (54.29)	55 (21.07)	1 (5.56)	8 (38.10)	29 (50.88)	136 (48.92)	10 (58.82)	1 (50)	0 (0)	N/A	1 (100)
**Vault fracture, *n*, %**	7 (43.75)	85 (34.69)	60 (22.99)	2 (11.11)	7 (33.33)	17 (29.82)	142 (51.08)	10 (58.82)	0 (0)	0 (0)	N/A	0 (0)
**Facial bone fracture, *n*, %**												
Mandible fracture	0 (0)	13 (5.31)	14 (5.36)	0 (0)	2 (9.52)	6 (10.53)	21 (7.55)	1 (5.88)	0 (0)	0 (0)	N/A	0 (0)
Maxilla fracture	3 (18.75)	73 (29.80)	50 (19.16)	1 (5.56)	8 (38.10)	8 (14.04)	26 (9.35)	1 (5.88)	0 (0)	0 (0)	N/A	0 (0)
Nose fracture	1 (6.25)	15 (6.12)	14 (5.36)	1 (5.56)	2 (9.52)	6 (10.53)	6 (2.16)	2 (11.76)	0 (0)	0 (0)	N/A	0 (0)
Orbit fracture	2 (12.5)	90 (36.73)	55 (21.07)	2 (11.11)	6 (28.57)	12 (21.05)	35 (12.59)	3 (17.65)	0 (0)	0 (0)	N/A	0 (0)
Zygoma fracture	1 (6.25)	71 (28.98)	48 (18.39)	2 (11.11)	11 (52.38)	10 (17.54)	28 (10.07)	1 (5.88)	0 (0)	0 (0)	N/A	0 (0)

## Data Availability

Data may be obtained from a third party and are not publicly available. The approving authority for data access was the Japan Trauma Care and Research (JTCR). Data are only available on request from the JTCR, and access requires appropriate ethical and governance clearances regarding use.
